# Blood Pressure Response and Pulse Arrival Time During Exercise Testing in Well-Trained Individuals

**DOI:** 10.3389/fphys.2022.863855

**Published:** 2022-07-11

**Authors:** Sondre Heimark, Ingrid Eitzen, Isabella Vianello, Kasper G. Bøtker-Rasmussen, Asgeir Mamen, Ole Marius Hoel Rindal, Bård Waldum-Grevbo, Øyvind Sandbakk, Trine M. Seeberg

**Affiliations:** ^1^ Department of Nephrology, Oslo University Hospital, Oslo, Norway; ^2^ Institute of Clinical Medicine, University of Oslo, Oslo, Norway; ^3^ Department of Smart Sensors and Microsystems, SINTEF Digital, Oslo, Norway; ^4^ Department of Health Science and Technology, Aalborg University, Aalborg, Denmark; ^5^ Kristiania University College, School of Health Sciences, Oslo, Norway; ^6^ Centre for Elite Sports Research, Department of Neuromedicine and Movement Science, Norwegian University of Science and Technology, Trondheim, Norway

**Keywords:** blood pressure response, continuous cuff-less measurement method, diastolic blood pressure (DBP), endurance athletes, pulse arrival time (PAT), systolic blood pressure (SBP)

## Abstract

**Introduction:** There is a lack of data describing the blood pressure response (BPR) in well-trained individuals. In addition, continuous bio-signal measurements are increasingly investigated to overcome the limitations of intermittent cuff-based BP measurements during exercise testing. Thus, the present study aimed to assess the BPR in well-trained individuals during a cycle ergometer test with a particular focus on the systolic BP (SBP) and to investigate pulse arrival time (PAT) as a continuous surrogate for SBP during exercise testing.

**Materials and Methods:** Eighteen well-trained male cyclists were included (32.4 ± 9.4 years; maximal oxygen uptake 63 ± 10 ml/min/kg) and performed a stepwise lactate threshold test with 5-minute stages, followed by a continuous test to voluntary exhaustion with 1-min increments when cycling on an ergometer. BP was measured with a standard automated exercise BP cuff. PAT was measured continuously with a non-invasive physiological measurements device (IsenseU) and metabolic consumption was measured continuously during both tests.

**Results:** At lactate threshold (281 ± 56 W) and maximal intensity test (403 ± 61 W), SBP increased from resting values of 136 ± 9 mmHg to maximal values of 219 ± 21 mmHg and 231 ± 18 mmHg, respectively. Linear within-participant regression lines between PAT and SBP showed a mean *r* ^2^ of 0.81 ± 17.

**Conclusion:** In the present study focusing on the BPR in well-trained individuals, we observed a more exaggerated systolic BPR than in comparable recent studies. Future research should follow up on these findings to clarify the clinical implications of the high BPR in well-trained individuals. In addition, PAT showed strong intra-individual associations, indicating potential use as a surrogate SBP measurement during exercise testing.

## Introduction

Blood pressure (BP) measurement is included as a regular component of exercise stress testing, in order to evaluate the physiological status of the individual and detect subclinical cardiovascular disease (CVD) (Schultz et al., 2012; Miyai et al., 2021). The normal BP response (BPR) to an increase in intensity during dynamic exercise includes a rise in systolic blood pressure (SBP) as well as decreasing total peripheral resistance (TPR), whereas the diastolic blood pressure (DBP) remains stable or reveals a slight decrease (Bjarnason-Wehrens and Predel, 2020). Exaggerated BPR to exercise has been reported as a prognostic factor for incident hypertension or cardiovascular disease in the general population (Schultz and Sharman, 2013; Caselli et al., 2019; Mariampillai et al., 2020). This so-called hypertensive response to exercise has been defined as an SBP ≥ 210 mmHg for men and ≥ 190 mmHg for women (Schultz and Sharman, 2013). However, these thresholds and the subsequent clinical interpretation of what should be regarded as a normal or abnormal BPR during exercise are under debate (Bauer et al., 2021), and The European Society of Cardiology states in its latest guideline that there is currently no consensus on what is to be defined as a ‘normal’ BPR during exercise (Williams et al., 2018). Thus, there is a strong need for information related to the clinical value of exercise-related BP in the general population (Bjarnason-Wehrens and Predel, 2020).

With regard to BPR to exercise in well-trained or athletic populations, data are even more sparse, and ambiguity is inherently even larger. Even though exercise testing plays a pivotal role in sports cardiology, few studies have evaluated the magnitude and distribution of exercise-induced BPR in athletic populations. Due to the high cardiac output achieved by athletes, the upper limit of ‘normal values’ for peak exercise SBP may differ from other populations. While an exaggerated exercise BP is increasingly regarded as a risk factor for cardiovascular disease in the general population (Schultz et al., 2017), the clinical importance of exercise BPR in athletes remains uncertain. A recent review by Richard et al. (2021) states that existent guidelines cannot be adapted when evaluating BPR in endurance-trained individuals and underlines that elevated SBP in this population might reflect adaptive responses to training rather than a pathological sign. Furthermore, comparison of results from the studies involving athletes that do exist is challenging, due to differences in reported exercise testing methods and protocols, BP measurement methods, and determinations of SBP at maximum or sub-maximum workloads (Pressler et al., 2018; Caselli et al., 2019; Bauer et al., 2020). Hence, a consensus on what is a normal BPR response to exercise in well-trained individuals, as well as direct causation linking high-graded exercise testing and SBP to pathology, is lacking.

Another obstacle in estimating BPR during exercise is caused by limitations in validated non-invasive measurement methods, which are usually cuff-based and may be impractical, intermittent, and give discomfort to patients (El-Hajj and Kyriacou, 2020). As a result, cuff-less BP monitoring with different bio-signal approaches has gained increasing attention (Pandit et al., 2020; Welykholowa et al., 2020). One of the signals regarded as promising for estimation of BP is the extraction of pulse arrival time (PAT) (Lee et al., 2019). PAT measured using an electrocardiography (ECG) sensor and a photo-plethysmography (PPG) sensor and is calculated as the time interval from an R-wave in an ECG signal to a fiducial point in a peripheral PPG waveform (Welykholowa et al., 2020). PAT is inversely related to pulse wave velocity (PWV) and the relationship between PWV and BP has been recognized since the late 19th century (Callaghan et al., 1984). When investigating BPR during exercise, a continuous cuff-less approach would be superior compared to cuff-based measurements, to avoid discomfort and inaccuracy caused by movement. However, PAT is a single parameter, and its relationship to different parameters of BP is not yet fully understood (Heimark et al., 2021). Still, the correlation coefficient (on an individual basis) between PAT and SBP in a recent review by Welykholowa et al. (2020) was shown to be *r* = 0.84 (range 0.42–0.98) in studies utilizing the ECG + PPG modality. Thus, it is reasonable to assess PAT as a potential cuff-less surrogate SBP measurement during exercise testing in athletes.

The present study aimed to investigate the BPR in a population of well-trained individuals during a lactate threshold and maximal exercise test on a cycle ergometer, utilizing both an auscultatory technique with an upper arm inflatable cuff with an integrated microphone designed for exercise, and a novel device aimed for cuff-less BP measurements to extract PAT as a potential surrogate BP measurement. The study will add to current knowledge on BPR during exercise testing in an athletic population by addressing the following two aims: 1) To investigate the systolic BPR during a lactate threshold and maximal cycle ergometer test in a population of well-trained male cyclists, and 2) To assess whether PAT measured with a novel cuff-less device can be used as a continuous SBP surrogate during exercise testing in the athletic population.

## Materials and Methods

### Participants

Well-trained male cyclists over 18 years of age, free of any chronic and cardiovascular disease and not under any form of pharmacological treatment, were eligible for inclusion. Candidates were recruited from cycling clubs in Oslo and surrounding areas. Qualifying performance criteria were; experience in high-intensity bike exercise and a minimum of 8 hours of exercise per week, of which a minimum of 5 hours of cycling. In line with the Helsinki declaration, all participants were informed about the test procedure and signed a written informed consent form before final inclusion. The participants were instructed to be fully recovered on the test day, which included avoiding training earlier on the same day and accomplishing only light training on the day before. They were further encouraged to ensure an adequate food and fluid intake on the day before and earlier during the test day, and to avoid intake of any food during the last hour and caffeine drinks the last 3 hours before the test. During the test, participants were shirtless, wearing only padded cycling tights and cycling shoes. If needed, the areas on which the electrodes were applied were shaved free of hair.

### Equipment

BP at rest and during the exercise test was measured utilizing an auscultatory technique with an upper arm inflatable cuff with an integrated microphone designed for exercise tests (Schiller BP-200+, Schiller AG, Baar, Switzerland). The auscultatory technique BP device was calibrated by certified service personnel the day before the testing started (Diacor, Oslo, Norway), and the following settings were used: max inflation pressure of 300 mmHg, and a deflation rate of 3 mmHg/s. This setup for measuring BP also consists of a 3-channel ECG sensor (Schiller Cardiovit AT-104 PC, Schiller AG, Baar, Switzerland) that were used by Schiller BP-200 + to identify heart cycles (QRS triggering) to provide more accurate BP measurements.

Respiratory variables were measured continuously during the exercise test using a metabolic analyzer (Cosmed K5, Cosmed srl, Rome, Italy, software Omnia 1.6.5) and data was automatically synchronized with the Schiller ECG data. Prior to each test, the metabolic analyzer was calibrated in accordance with the user guide. This calibration included flow calibration by a 3 L calibration syringe (Cosmed ref C00600.01.11), CO_2_ zeroing (shrubbery) and reference gas calibration, O_2_ = 16.0% and CO_2_ = 4.0% (CareFusion, Yorba Linda, CA, United States). Finally, blood lactate concentration (BLa) was measured with Lactate Scout, a portable analyzer (Lactate Scout+, EKF Diagnostics, Cardiff, United Kingdom).

A Garmin sports watch (Garmin Forerunner 920XT, Olathe, KS, United States) with a chest belt for measuring heart rate (HR) was used to give real-time measures to the investigators during the whole protocol. In addition, the Garmin device provided a master timeline for the protocol.

A prototype, wearable device, IsenseU (SINTEF, Oslo, Norway), capable of measuring a one-channel ECG, PPG, impedance cardiography, and movements (3D-acceleration, 3D-angular rate) in the chest area was used to extract PAT from the ECG signal to the PPG at chest level. The PPG sensor was mounted on the case (12.5 cm × 4.5 cm) facing the body and ECG was measured using two standard electrodes placed on the anterior chest wall. Details on the devices have been published previously (Seeberg et al., 2017). An upgraded version with a higher sampling rate of 1,000 Hz was used in the present study. Raw signals were inspected in real-time via bluetooth connection to a custom-made software (SINTEF, Oslo, Norway).

### Study Protocol

#### Set-Up and Subject Preparation

**FIGURE 1:**
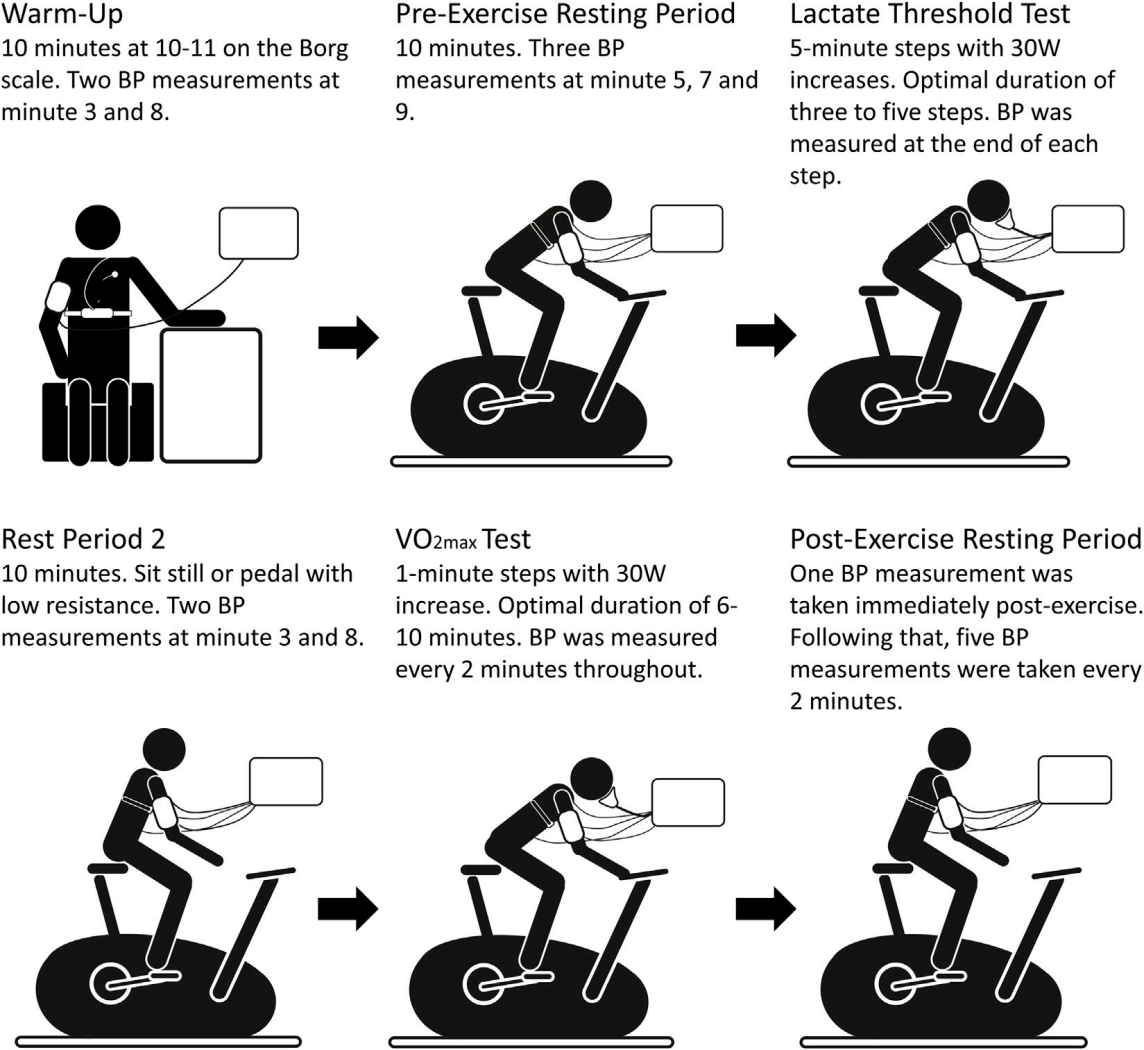
Illustration of the test protocol. BP, Blood pressure.

The test protocol is presented in Figure 1. All tests were performed in an exercise laboratory (Kristiania University College) on a cycle ergometer and comprehended a lactate threshold test followed by a maximal intensity test. The test protocol used in the present study is identical to the protocol utilized by the Norwegian Olympic and Paralympic Committee and Confederation of Sport at the time of planning the study. Participants’ anthropometric measurements (birth date, height, weight, body mass index (BMI), and body fat percentage) were recorded prior to the test. The cycle ergometer (Lode Excalibur Sport, Lode B.V., Groningen, Netherlands) was calibrated 3 months before testing by certified personnel (Timik, Oslo), and adjusted prior to each test subject in accordance with their anthropometric data. The temperature in the laboratory was 20.1 ± 0.9°C.

The BP cuff was placed on the upper left arm of the subject (at heart level), with bladder size adjusted to the arm circumference and with the microphone positioned on the brachial artery. For some participants, a towel was placed between the lower arm and the handlebar so that the subject could be more comfortable. The IsenseU sensor was kept fixed on the chest by an elastic chest belt with three standard ECG electrodes positioned on the upper chest, as described by Seeberg et al. (2017). The Garmin chest belt HR monitor was positioned just below the IsenseU sensor. Continuous HR was measured throughout the whole test protocol. The 3-lead ECG was fitted with three standard electrodes; two were placed below the clavicle at the right and left upper part of the chest and the third at the lower left part of the chest.

#### Pre-Exercise Resting Period

Prior to the exercise test, the participants rested for 10 min seated in a chair while synchronized continuous data collection was initialized for the IsenseU, Garmin watch, and the 3-lead ECG. Resting BP was measured at the 5th, 7th, and 9th minute. The lowest recorded value of the three was defined as the resting BP value. After the last resting BP measurement, BLa at rest was recorded.

#### Warm-Up

After rest measurements, the participants performed a 10-minute warm-up period on the cycle ergometer at a controlled self-selected intensity corresponding to a rate of perceived exertion (RPE) of 10–11 on the Borg Scale (Borg, 1982). Two BP measurements were recorded at the 3rd and 8th minute. The warm-up period was followed by a short break, during which the mask for the metabolic recording was placed and checked for leakage (Hans Rudolph 7450 Series V2 mask with 2600 non-rebreathing *Y*-valve; Hans Rudolph Inc, Shawnee, KS, United States).

#### Lactate Threshold Test

Oxygen uptake was recorded throughout the test. The starting intensity of the threshold test was set between 60 and 90 W below the presumed lactate threshold at the nearest predefined unit with 30 W intervals. It was assumed that the participants knew their approximate anaerobic threshold load (W). In case this was not known, the starting load was at RPE of 11, with HR and RPE feedback from the warm-up period. Cadence was kept at 85-95 revolutions per minute (RPM) throughout the test.

The lactate threshold test consisted of 5-minute steps with an incremental increase in the workload of 30 W and an optimal duration between three and five steps. The BLa was measured by a finger capillary sample at the last 15 s of each increment. In cases where the BLa had reached a level between 3 and 4 mmol/L, the subsequent incremental increase was 15 W instead of 30 W. When the BLa reached a level above or close to 4 mmol/L the test was concluded. The reason for the conclusion at levels close to 4 mmol/L was that the addition of one more increment would spike the BLa significantly above 4 mm/L and potentially disrupt performance during the subsequent maximal incremental test. BP was measured starting 1 min and 40 s prior to the end of each incremental step and without pause in the test, in order to have an adaptation of the BP to the exercise intensity.

#### Rest Period two

After completion of the threshold test, the mask was taken off and all participants had a resting period of 10 min. Participants could decide whether to sit still on the bike or to pedal at a very low intensity to avoid an undesired stiffening of the legs. Two BP measurements were taken at the 3rd and 8th minute.

#### VO_2max_ Test

At the end of the break, the mask to measure the metabolic consumption was re-mounted. The VO_2max_ test started at the same intensity as the threshold test in cases where the anaerobic threshold was reached at the 4th or 5th incremental step. Otherwise, it started at 30 W lower if the threshold was reached earlier, or at 30 W higher if the threshold was reached later. The optimal duration of the VO_2max_ test was between 6 and 10 min. The incremental steps had a duration of 1 min with an increase of 30 W. The cadence was kept at 85–95 RPM throughout the test. The test continued until exhaustion was reached by the subject; defined as a voluntary interruption or by the cadence falling below 60 RPM, despite a strong encouragement to hold on to the given intensity. After completion, the mask was removed, and the metabolic recording turned off. BLa was measured 1-minute post-exercise. BP measurements during the test were recorded starting from the first minute of exercise at every second minute until exhaustion. IsenseU-data, metabolic capacity, and HR were recorded throughout the test.

#### Post-Exercise Resting Period

A post-exhaustion BP measurement was taken immediately after completion of the VO_2max_ test when the subject was recovering seated on the cycle ergometer. Following the BP measurement, participants were seated on a chair while five post-exercise BP measurements were taken every second minute for 10 min.

### Data Processing and Statistical Analyses

#### Synchronization of Sensor Data

First, the IsenseU-data was attached to the master-timeline (i.e. the time of the Garmin watch) by using manually noted times for the start/stop of the sensor. Then data from the metabolic analyzer (which was automatically synchronized with the Schiller ECG data), was synchronized to the master-timeline by accomplishing maximum correlation of RR-peaks in the two ECG signals (IsenseU and Schiller). Finally, the open-source software platform Activity Presenter, a software module created to simplify the process of visualizing, synchronizing, and organizing data and video from multiple sources (Albrektsen et al., 2022), was used to fine-tune and verify correct synchronization.

#### Performance Characteristic and Variation of Physiological Measurements Between Participants

**FIGURE 2:**
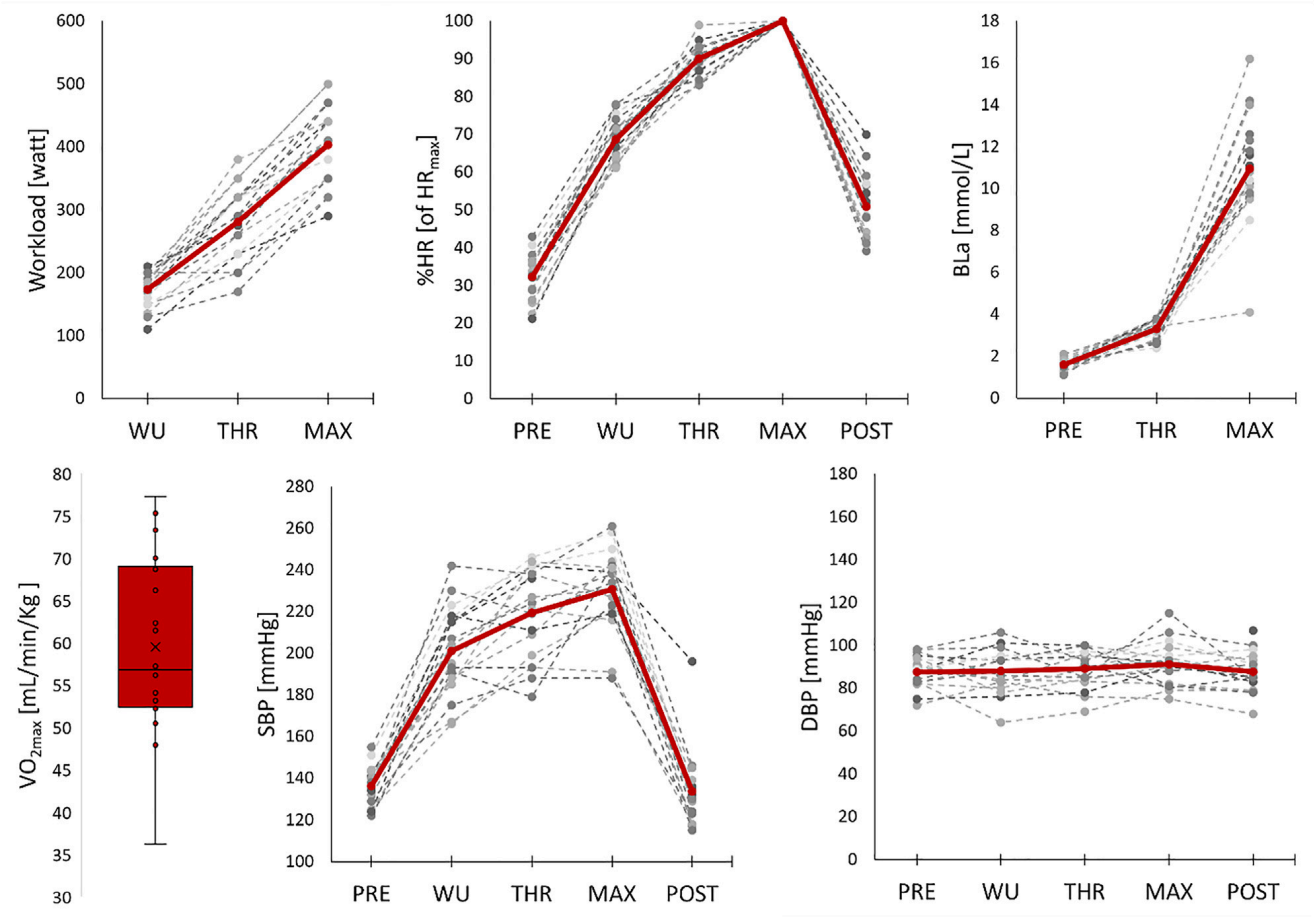
VO_2max_ is presented as a box and whisker plot with median, interquartile range, and minimum and maximum value, otherwise Individual (dotted lines) and mean (red line) values for workload, percentage of maximal heart rate (%HR), blood lactate (BLa), systolic blood pressure (SBP) and diastolic blood pressure (DBP) during the protocol. PRE = values at rest before the cycling, WU = values during warm-up, THR = values at the threshold, MAX = values at maximal intensity (except for BLa which was measured directly after the maximal intensity), POST = values measured 10 min after the test.

To be able to compare data across participants, five characteristic physiological states were defined and the corresponding data at those points were extracted from the dataset (presented in Figure 2):

• PRE: value at rest before the cycling. The measurement point with the lowest SBP was used with the corresponding HR.
• WU: value during warm-up. The last BP measurement (after 8 min) was used as the subject-dependent intensity was adjusted and the physiological parameters were stabilized to the intensity demand.
• THR: values at 4.0 mmol/L as an indication of lactate threshold. The last completed BP measurement with the corresponding HR and watt values before passing BLa of 4.0 mmol/L was extracted.
• MAX = Watt at the last completed stage in the maximal test and maximal HR reached in the test. For the BP measurements, the point with the highest completed valid measurement was chosen. It should be noted that this was lower than the maximal W reached during the max-test because the BP measurement took longer than each increment and it was difficult to obtain valid measurements during maximal intensity. BLa was measured directly after the maximal test.
• POST = SBP with the corresponding HR measured 10 min after completion of the maximal test.

#### Calculation of PAT and SBP-Corresponding PAT Values

PAT was calculated for each cardiac cycle from the R-peak in the ECG signal to the foot in the PPG signal recorded from the skin vasculature at chest level (Heimark et al., 2021). The PAT values were filtered by applying a moving median filter with a window size of 30 cycles, only keeping PAT values within 20% of the median of the window. Subsequently, a second median filter was applied to calculate the median PAT value from 10 cycles prior to and after the corresponding SBP value. SBP values were noted manually at the time of recording and synchronized to IsenseU as described earlier. To ensure that the PAT was not calculated too far away from its corresponding SBP measurement, PAT values calculated more than 20 s apart from the SBP measurement were discarded. If less than 5 valid PAT values existed in the window, the PAT measurement was discarded. DBP was not considered in the present analysis due to a lack of relationship between the two variables (Heimark et al., 2021).

#### Participant Selection

All participants (*n* = 18) were included in the analysis of performance and variation of physiological response during the lactate threshold and maximal dynamic exercise test. For the analysis of PAT, three subjects had to be excluded due to a low signal-to-noise ratio in the PPG, and thus only 15 were available for analyses of the relationship between PAT and SBP.

#### Statistical Analyses

Raw data processing and statistical analyses were performed using the Python programming language with these packages; NumPy (1.18.1), SciPy (1.4.1), NeuroKit2 (0.0.32), Pandas (1.3.3), and Matplotlib (3.1.1) (Hunter, 2007; McKinney, 2010; Harris et al., 2020; Virtanen et al., 2020; Makowski et al., 2021). Prior to the linear regression analysis, all PAT SBP pair outliers were filtered in the following way: If the probability of the pair occurring was less than 2.5% based on the normal Gaussian distribution from all samples from the same subject, the measurement pair was considered an outlier and removed from the data analysis. The 2.5% cut-off was selected due to increasing levels of noise in both the raw signals from the IsenseU and the BP cuff with increasing levels of exercise intensity. The relationship between PAT and SBP was analyzed using linear regression for each subject. All valid measurement pairs for each participant throughout the test protocol, including rest periods and warm-up, were used. For the performance characteristics of the five defined physiological states, mean and standard deviations were calculated.

## Results

### Participant Characteristics

General characteristics of the 18 participants were; mean age of 32.4 ± 9.4 years, height of 182.7 ± 6.6 cm, body mass of 75.4 ± 8.2 kg, BMI of 22.6 ± 1.5 kg/m^2,^ and a body fat percentage of 9.3 ± 3.9%. The participants rode an average of 14,130 ± 7240 km/year and had 7.9 ± 4.8 years of experience in active cycling.

### Performance Characteristics and Blood Pressure Response

**FIGURE 3:**
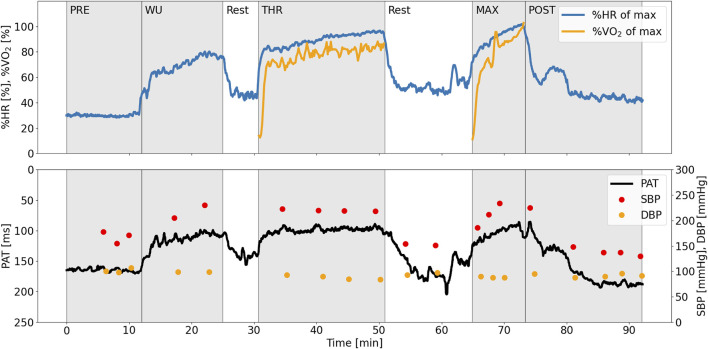
Physiological measurements during the experimental protocol, exemplified with data from one typical subject. PAT is inverted on the *y*-axis to better visualize the co-variation with SBP. PAT, pulse arrival time (ms); SBP, systolic blood pressure (mmHg); DBP, diastolic blood pressure (mmHg); HR, heart rate (beats per minute).

To highlight the test protocol, physiological measurements for one typical participant are displayed in Figure 3. Performance parameters and BP response extracted from the pre-exercise resting period (PRE), warm-up (WU), at threshold (THR), at maximal intensity (MAX, except for BLa which was measured directly after the maximal intensity), and 10 min after the test (POST) are presented in Table 1. In brief, the participants had a mean VO_2max_ of 63 ± 10 ml/min/kg and maximum workload in the maximal performance test at 403 ± 61 W. Resting BP was 136/88 ± 9/7 mmHg and SBP at MAX was 231 ± 18 mmHg. Individual data and mean values for performance and BP characteristics for the participants during the protocol are given in Figure 2.

**TABLE 1:** Mean values and standard deviation for workload, VO_2_, %VO_2_, HR, %HR, systolic blood pressure (SBP), diastolic blood pressure (DBP), and blood lactate (BLa) during the protocol.

	PRE	WU	THR	MAX	POST
	Mean (SD)	Mean (SD)	Mean (SD)	Mean (SD)	Mean (SD)
Workload [Watt]		174 (28)	281 (56)	403 (61)	
VO2 [ml/min/kg]			49 (9)	63 (10)	
%VO2 [of VO2max]			82 (12)	100 (0)	
HR [bpm]	59 (11)	125 (12)	164 (10)	182 (9)	93 (18)
%HR [of HRmax]	32 (6)	69 (5)	90 (4)	100 (0)	51 (8)
SBP [mmHg]	136 (9)	201 (21)	219 (21)	231 (18)	134 (18)
DBP [mmHg]	88 (7)	88 (10)	89 (9)	91 (10)	88 (9)
BLa [mmol/L]	1.6 (0.4)		3.3 (0.5)	11.0 (2.5)	

PRE = values at rest before the cycling, WU = values during warm-up, THR = values at the threshold, MAX = values at maximal (except for Bla which was measured directly after maximal intensity), POST = values measured 10 min after the test

Table 2 shows the results from the linear regression between SBP and PAT. The mean *r* ^2^ of all individual regression analyses was 0.81 ± 0.17 with a mean of individual regression slopes of −0.72 ± 0.37 ms/mmHg.

**TABLE 2:** Linear regression between systolic blood pressure (SBP) and pulse arrival time (PAT) for participants with valid pulse arrival time (PAT) measurements. The *p* value indicates the test of the null hypothesis that the coefficients are equal to zero.

Participant number	*r* ^2^	Slope (ms/mmHg)	*p* value	Number of measurement pairs removed based on poor signal quality
1	0.91	−0.51	0.001	10/17
2	0.69	−0.66	0.005	13/22
3	0.97	−0.65	< 0.001	1/20
4	0.84	−1.09	< 0.001	3/22
5	0.32	−0.18	0.014	2/20
6	0.90	−0.79	< 0.001	2/20
7	0.93	−0.76	< 0.001	6/20
8	0.93	−1.00	< 0.001	0/19
9	0.77	−1.59	< 0.001	0/20
10	0.86	−0.73	< 0.001	1/20
11	0.82	−0.44	< 0.001	3/23
12	0.90	−0.93	< 0.001	8/21
13	0.64	−0.69	< 0.001	4/22
14	0.82	−0.59	< 0.001	1/15
15	0.91	−0–86	< 0.001	3/22
Mean ± SD	0.81 ± 0.17	−0.72 ± 0.37		
	*n* = 15

## Discussion

At present, few studies have described the BPR in well-trained individuals and athletes, and it is not known whether an exaggerated BPR represents a warning sign, or rather is an expression of adaptive responses to training in these populations (Richard et al., 2021). The primary aim of this study was, therefore, to add to the current knowledge by investigating the BPR during a maximal cycle ergometer test in well-trained male cyclists. Our results indicate, similarly to previous studies, that the systolic BPR during maximal aerobic exercise in well-trained subjects is exaggerated compared to normative values from a general population. Notably, the SBP at peak aerobic intensity from our cohort was even higher than in recent similar studies. Furthermore, cuff-less approaches are suggested to overcome the current limitations of cuff measurements during exercise testing. Thus, as a secondary aim, we investigated PAT as a potential non-invasive cuff-less measurement method. Here, our results strengthen previous findings of strong associations between PAT and exercise SBP on an individual level.

### Blood Pressure Response in Well-Trained Individuals

In our study, the mean (SD) value for SBP at peak aerobic exercise was 231 (18) mmHg, with a mean difference from baseline of 95 mmHg. There is no consensus on the exact definition for what should be regarded as an exaggerated BP response to exercise in general, even though SBP at peak aerobic exercise exceeding 210 mmHg for men and 190 mmHg for women has frequently been reported as exaggerated (Schultz and Sharman, 2013; Sabbahi et al., 2018; Percuku et al., 2019). An alternative suggested cut-off in several studies is a difference of 60 mmHg between baseline (resting) and peak SBP for men and 50 mm Hg for women (Percuku et al., 2019). Caselli et al. (2016) tried to accommodate the shortcoming of reference values specifically for the athletic population by assessing BPR in highly trained Olympic athletes and suggested a cut-off for males of 220 mmHg peak SBP. From recent similar studies listed in Table 3, Pressler et al. (2018), Caselli et al. (2019) and Bauer et al. (2020) reported mean peak aerobic SBPs somewhat lower than in the present study, but close to the suggested cut-off values in athletes. As a consequence, a large number of athletes still have an exaggerated systolic BPR. Importantly, the data from Caselli et al. (2019) are extracted from a cohort also including 27% women, and separate values for men were not given. As men consistently are reported to reveal a more pronounced SBP response than women (De Buyzere and Rietzschel, 2018; Song et al., 2020), the value for men-only would likely be higher. Comparably high maximum dynamic exercise BPR to our findings has previously been reported in similar populations. Karjalainen et al. (Table 3) reported a peak aerobic SBP of 228 (16) mmHg in elite male orienteers and long-distance runners aged 26 (±3) (Karjalainen et al., 1997). Compared with age and gender-matched normative data in apparently healthy general population cohorts, Hedman et al. (2020b) reported a peak SBP during exercise test of 202 (22) mmHg and Sabbahi et al. (2018) 179 (20) mmHg, with corresponding differences from baseline of 74 and 56 mmHg, respectively. In sum, our data indicate an exaggerated BPR in well-trained individuals compared to the general population and reflect an even higher peak SBP than in recent comparable studies on athletic populations.

**TABLE 3:** Overview of similar studies.

Study	Cohort, number of participants, age (SD)	Peak aerobic SBP (SD), mmHg	Maximal workload (SD), Watt	Difference from baseline, mmHg	Exercise method	BP measurement method	Test protocol
Present study	Well-trained male cyclists, 18, 32 (9.4)	231 (18)	403 (61)	95*	Cycle ergometry	Automated electronic exercise cuff	Threshold W followed by 30 W increases every 1 min
Bauer et al. (2020)	Male professional handball and hockey athletes, 142, 26 (5)	197 (20)	351 (79)	74 (20)	Cycle ergometry	Automated electronic exercise cuff	100 W followed by 50 W increases every 2 min until exhaustion
Pressler et al. (2018)	Professional athletes, 2419 (663 female), 26 (12)	204 (22)	305 (59)	80 (20	Cycle ergometry	Manual sphygmo-manometry	Varying; usually starting load of 50-100 W with 20-50 W increases and 3-minute durations
Caselli et al. (2019)	Male and female professional athletes, 141, 26 (6)	208 (22)	262 (61)	87*	Cycle ergometry	Manual sphygmo-manometry	0.5W/Kg with increases of 0.5W/Kg every 2 min
Karjalainen et al. (1997)	Male orienteer and long distance runners, 32, 26 (3)	228 (16)	333 (27)	97*	Cycle ergometry	Manual sphygmo-manometry	50 W followed by 50 W increase every 3 min

SBP, systolic blood pressure. SD, standard deviation. W, watt. *SD unknown

One possible explanation for the observed high peak SBP values in our study compared to recent comparable studies is that our participants had a higher mean age. It has been shown that peak exercise SBP increases steadily with increasing age (Hedman et al., 2020b). However, in the study by Karjalainen et al., which demonstrated similarly high peak aerobic SBP, participant age was lower compared to our study. Another possible explanation is that cyclists compared to other athletes are shown to have a larger BPR during a maximal cycle ergometry test (Richard et al., 2021), and none of the other studies included cyclists-only. It is further important to bear in mind that there is no accepted gold standard exercise BP measurement method, which may impact the comparability across studies, particularly if the number of participants is small, as in our cohort. Another variable which differentiated our results from the afore discussed studies, was the higher achieved workload at peak aerobic exercise, presented in Table 3. All four comparable studies had test protocols that consisted of cycle ergometry. Pressler et al. (2018), Caselli et al. (2019) and Bauer et al. (2020) had considerably lower achieved workloads. Also in the study by Karjalainen et al. (1997), which had the most comparable BPR, the athletes achieved lower a maximal workload; of 333 (27) W. Whether the maximum workload is causal towards the higher peak SBPs by physiological explanations such as differences in TPR, or a result of well-trained cyclists performing a cycle-ergometer test, is uncertain. However, novel approaches in interpreting SBP response to peak aerobic exercise include indexing maximum SBP to max achieved workload (Hedman et al., 2020a).

Interestingly, the suggested alternative cut-off of 60 mmHg from rest to peak aerobic exercise is exceeded not only in our study but also in all four comparable studies on athletes as well as the normative data. The uncertainty of what should be regarded as a potentially dangerous exaggerated response is further complicated by the variance in prevalence of an exaggerated BPR between studies, not only due to different definitions but also because the BRP must be seen in relation to the characteristics of different study populations (Schultz and Sharman, 2013). It is further crucial to interpret values from the level of exercise intensity. Both our findings and results from comparable studies (Table 3) suggest that a cut-off of 60 mmHg increase from rest to peak SBP for exaggerated BP response may be inaccurate. Another factor that must be taken into consideration when comparing findings between studies is how they have defined measurements at “rest”. Body position may affect the measurements (Eser et al., 2007), and the white-coat effect should also be considered. For athletes, in particular, exercise testing may entail expectations of performance, which can lead to anxiety and an elevated stress level (Ford et al., 2017) which may influence their baseline data; in this context resting BP.

Although there is a growing body of studies indicating that an exaggerated BPR in athletes is a matter of physiological adaptation, there is a lack of longitudinal studies assessing if there is an increased risk of hypertension or cardiovascular disease. Caselli et al. (2019) showed that athletes with an exaggerated BPR compared to normal BPR to maximal cycle ergometry [max SBP 208 (22) mmHg vs. 185 (20) mmHg achieving maximal workloads of 262 (61) W vs. 257 (62) W] had a 3.6-fold hazard ratio of incident hypertension after 6.5 ± 2.8 years of follow up. These results highlight the need for future studies on athlete populations to define cut-off values and risk assessment.

### Is PAT a Potential Non-Invasive, Continuous Surrogate Systolic Blood Pressure Measurement in Athletic Populations?

Our results demonstrated that, on an individual level, PAT has a strong association with SBP in well-trained individuals during a threshold and VO_2max_ test. This has not previously been assessed in an athletic population with corresponding exercise intensities. PAT has gained increasing interest in non-invasive, cuff-less BP monitoring to overcome cuff limitations. Gold standard BP measurements during exercise are limited to the invasive method, which is not ethically justifiable in routine exercise testing or even in most research settings. Cuff-based methods, either manually or electronically by using a microphone to detect Korotkoff sounds, are considered acceptable but remain unable to produce high precision non-invasive measurements. Cuff-based methods are further limited due to intermittent sampling and distortion caused by noise and motion artifacts. Previous studies have, similar to our findings, demonstrated that PAT is strongly correlated to SBP on an individual level during dynamic exercise (Wibmer et al., 2015; Heimark et al., 2021). However, the need for calibration against a cuff-measurement to correct for the unknown length of the pulse wave propagation in addition to other individual factors is still an unresolved limitation in PAT-based approaches. Wibner et al. (2015) achieved comparable coefficients of determination to our results using regression analysis in 18 patients referred to cardiopulmonary exercise testing, with a mean *r* ^2^ (SD) of 0.80 (0.22) vs. 0.81 (0.17) in our study. Wibner et al. further translated PAT to absolute BP values using multipoint calibration and achieved a Bland Altmann bias of –0.3 (12.4) mmHg with limits of agreement from –24.7 to 24.1 mmHg compared to the reference exercise BP cuff, which was considerably better than simultaneously measured continuous volume clamp method [bias 14.0 (28.5) mmHg)]. Thus, our results indicate that PAT may be a feasible continuous SBP surrogate measurement also in an athletic population. However, a major limitation to overcome, in addition to the need for at least one static calibration to adjust for individual offsets, is the significant between-individuals variation in the PAT/SBP slope. Wibner et al. were able to produce accurate SBP values for comparative purposes by multipoint calibration; however, this is not a practical approach in everyday use. Future research should focus on methods to predict the individual PAT/SBP slope.

Regarding DBP, no meaningful association with PAT was observed as DBP during dynamic exercise changed very little, which is expected from the underlying physiological adaptations. Similar indications have been reported in previous studies assessing PAT during dynamic exercise (Wibmer et al., 2015; Heimark et al., 2021). Although many studies report strong correlations between PAT and both SBP and DBP, we believe that PAT as a single parameter cannot be generalized to both SBP and DBP across various hemodynamic states.

It is an ongoing debate whether confounding of the pre-ejection period (PEP) limits the application of PAT as a BP surrogate measurement. PEP is defined as the electromechanical time delay from the electrical onset of the systole (observable as the R-peak in an ECG signal) to the actual opening of the aortic valve and true onset of the pulse wave propagation in the arterial tree, defined as pulse transit time (PTT). The PEP may not vary with the same magnitude and direction as the PTT and corresponding change in BP (Pour Ebrahim et al., 2019). However, during dynamic exercise, PEP is previously shown to display an intensity-dependent decrease from rest to exercise (Michael et al., 2017), potentially minimizing the confounding effect during exercise testing compared to non-exercise settings of BP measurement. Our study is limited to PAT only, and the potential confounding role of PEP should be clarified in future studies.

## Limitations

The main limitations of the present study are the small study sample containing only males, and the lack of a control group. Most investigations examining exaggerated BPR during exercise are derived from Caucasian middle-aged men, and there is a lack of studies including younger individuals and specifically athletes and/or well-trained individuals. There is further a lack of studies including women. Our study only partly addresses this bias, as our material consists of Caucasian men with a mean age of 32.4 years and a VO_2max_ of 63 (10) ml/min/kg. In addition, peak aerobic SBP suffers from varying test protocols with different methods of BP measurements across and within studies. Thus, the aforementioned limitations warrant caution when interpreting the results and drawing conclusions in the context of data from comparable studies. An important consideration when assessing the BPR to exercise is the change in BP from baseline values, of which the validity is dependent on true baseline or resting values. We observed in the present study, despite sitting rest for 5 min prior to baseline measurements, unreasonably high resting BP values, which was the reason for choosing the lowest value. The most likely explanation for this high resting BP is the anticipation of the subsequent VO_2max_ test. Our PAT analysis was limited by significant amounts of noise in the PPG signal, which was attributable to movement artifacts and clipping of high amplitude PPG waveforms during exercise. However, strict criteria were applied to measure PAT from valid signals during high noise periods. A major challenge in future developments in the PPG-sensor technology and signal processing is improvements to account for noise.

## Conclusion

The present study adds to existing data on the BPR in well-trained populations. The results suggest an exaggerated BPR compared to normative cut-off values and reveal a higher SBP at peak aerobic exercise compared to most similar studies. Our findings indicate that athletes may have different cut-off values than less trained populations, which could be a result of physiological adaptations. However, there is a need for more data to determine reliable cut-off values in addition to considering any possible risk factors associated with exaggerated SBP responses in athletic populations. Furthermore, the results suggest that PAT may be used as a potential non-invasive and cuff-less SBP surrogate measurement on an individual level. However, a measurement device with a more robust signal-to-noise ratio is required, and the varying individual relationship between PAT and SBP remains a challenge for future work.

## Data Availability

The raw data supporting the conclusions of this article will be made available by the authors, without undue reservation.
